# Supplementary material for: Polyketide synthase gene clusters in the ericoid mycorrhizal fungus Oidiodendron maius: genome-wide prediction, expression profiling, and functional characterization of knockout mutants
Source: Front Plant Sci. 2026 Sep 16;17:1924430. doi: 10.3389/fpls.2026.1924430 (PMC13623903; doi:10.3389/fpls.2026.1924430)
Supplement: Supplementary file 1 [file SupplementaryFile1.zip › Supplementary Materials/Supplementary Folder 1.zip!/Oidma1_AssemblyScaffolds_Repeatmasked/knownclusterblast/cluster27/jgi.p_Oidma1_43492_mibig_hits.html]

| MIBiG Protein | Description | MIBiG Cluster | MiBiG Product | % ID | % Coverage | BLAST Score | E-value |
| --- | --- | --- | --- | --- | --- | --- | --- |
| ctg1\_orf003 |  | BGC0000685 | Brassicicene\_C\_biosynthetic\_gene\_cluster | 38 | 82.722513089 | 228 | 9.7e-60 |
| ADI24956.1 | GsfD | BGC0000070 | Griseofulvin\_biosynthetic\_gene\_cluster | 26 | 92.4083769634 | 147 | 3.7e-35 |
